# Escore VExUS na Alta Hospitalar como Preditor de Readmissão em Pacientes com Insuficiência Cardíaca Agudamente Descompensada: Estudo de Coorte

**DOI:** 10.36660/abc.20230745

**Published:** 2024-05-23

**Authors:** Paulo Maciel Rinaldi, Marcos Frata Rihl, Márcio Manozzo Boniatti

**Affiliations:** 1 Universidade Federal do Rio Grande do Sul Porto Alegre RS Brasil Universidade Federal do Rio Grande do Sul, Porto Alegre, RS – Brasil; 2 Faculdade de Medicina UNISINOS São Leopoldo RS Brasil Faculdade de Medicina da Universidade do Vale do Rio dos Sinos (UNISINOS), São Leopoldo, RS – Brasil; 3 Hospital de Clínicas de Porto Alegre Porto Alegre RS Brasil Hospital de Clínicas de Porto Alegre, Porto Alegre, RS – Brasil

**Keywords:** Insuficiência Cardíaca, Readmissão do Paciente, Qualidade de Vida

## Abstract

**Fundamento:**

A congestão venosa residual é um dos principais contribuintes para a readmissão de pacientes com insuficiência cardíaca, e o escore de ultrassonografia de excesso venoso (VExUS) é uma ferramenta potencialmente útil para avaliar a congestão sistêmica.

**Objetivos:**

O objetivo do presente estudo foi investigar a associação entre o escore VExUS antes da alta hospitalar em pacientes com insuficiência cardíaca e o risco de readmissão por insuficiência cardíaca agudamente descompensada (ICAD) em até 90 dias após a alta.

**Métodos:**

O presente estudo de coorte prospectivo envolveu adultos com sinais e sintomas de ICAD, fração de ejeção do ventrículo esquerdo de 40% ou menos (insuficiência cardíaca com fração de ejeção reduzida), sintomas de classe funcional II a IV da New York Heart Association e evidência clínica de congestão venosa necessitando de diuréticos intravenosos. Momentos antes da alta, realizamos avaliação do escore VExUS. O desfecho primário foi um desfecho composto de readmissão ou visitas de emergência devido à ICAD dentro de 90 dias após a alta hospitalar. A significância estatística foi estabelecida em p < 0,05.

**Resultados:**

A coorte foi composta por 49 indivíduos, dos quais 11 (22,4%) apresentaram o desfecho primário. Na alta, 34,7% dos participantes tiveram escore VExUS de 2 ou 3. Os pacientes com VExUS de 2 e 3 tiveram maior proporção do desfecho primário quando comparados aos pacientes com VExUS de 0 (35,3% versus 9%, p = 0,044).

**Conclusões:**

Uma proporção significativa de pacientes com insuficiência cardíaca com fração de ejeção reduzida admitidos por ICAD apresentou sinais clínicos e ultrassonográficos de congestão residual na alta. Pacientes com escore VExUS de 2 ou 3 no momento da alta hospitalar apresentaram maior risco de readmissões ou visitas de emergência por ICAD após 90 dias.

## Graphical abstract

**Figura Central:**
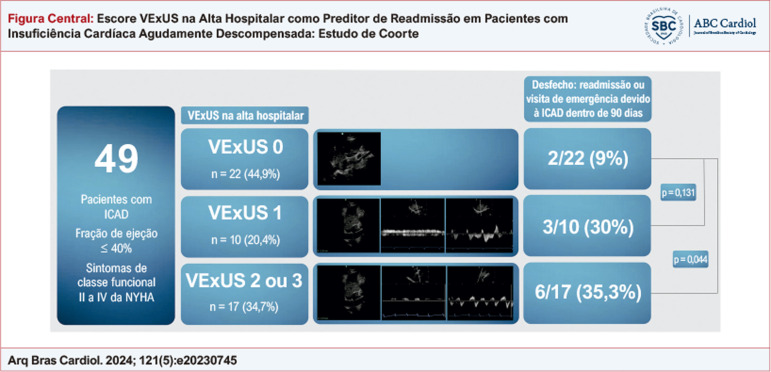
: Escore VExUS na Alta Hospitalar como Preditor de Readmissão em Pacientes com Insuficiência Cardíaca Agudamente Descompensada: Estudo de Coorte ICAD: insuficiência cardíaca agudamente descompensada; NYHA: New York Heart Association; VExUS: escore de ultrassonografia de excesso venoso.

## Introdução

A insuficiência cardíaca (IC) é uma condição altamente prevalente em todo o mundo, afetando mais de 64 milhões de pessoas e sendo responsável por uma proporção significativa de hospitalizações e readmissões.^1,2^ Infelizmente, até 50% dos pacientes são readmitidos dentro de 6 meses após a hospitalização inicial por IC,^3^ o que pode afetar substancialmente sua qualidade de vida.^4^

Os sinais e sintomas relacionados à congestão estão entre as causas mais comuns de hospitalização por IC e subsequentes readmissões,^5^ destacando a importância da congestão não resolvida após insuficiência cardíaca aguda descompensada (ICAD) como um dos principais contribuintes para taxas mais altas de readmissão.^6^ Consequentemente, o manejo da congestão clínica tem sido, há muito tempo, um dos principais objetivos da hospitalização.^7^ Entretanto, dados de registros revelam que cerca de 40% dos pacientes recebem alta apesar de sintomas persistentes de IC.^8,9^ Além disso, pressões de enchimento cardíaco elevadas podem existir sem congestão clínica, ressaltando o papel das anormalidades hemodinâmicas subclínicas na fisiopatologia da IC. Isso reforça a necessidade de uma avaliação abrangente do estado volêmico para otimizar o manejo do volume em pacientes com ICAD.^10^

O escore Efficacy of Vasopressin Antagonism in Heart Failure: Outcome Study with Tolvaptan (EVEREST) é uma ferramenta clínica usada para avaliar a congestão e orientar a terapia de descongestão em pacientes com ICAD.^10,11^ No entanto, as evidências atuais sugerem que os métodos tradicionais de avaliar a congestão, como a radiografia de tórax e a avaliação clínica, podem ter a precisão limitada. A ultrassonografia pulmonar emergiu como uma ferramenta promissora para avaliar a congestão pulmonar, com maior precisão que os métodos tradicionais.^12,13^ Em particular, foi demonstrado que a presença de linhas B na ultrassonografia pulmonar prediz um maior risco de readmissão ou morte em pacientes com IC.^14^ Outro método para avaliar a congestão sistêmica é o escore de ultrassonografia de excesso venoso (VExUS), que combina dilatação da veia cava inferior (VCI) e Doppler de onda pulsátil da veia hepática, porta e intrarrenal.^15^ Embora o escore VExUS tenha ganhado popularidade na avaliação da IC, sua utilidade para orientar a terapia ou prever desfechos permanece incerta. Portanto, o presente estudo visou investigar a associação entre o escore VExUS antes da alta hospitalar entre pacientes com insuficiência cardíaca com fração de ejeção reduzida (ICFEr) e o risco de readmissão ou visitas de emergência por ICAD dentro de 90 dias após a alta.

## Métodos

### População do estudo

O presente estudo de coorte prospectivo com amostragem por conveniência incluiu pacientes com 18 anos ou mais que apresentavam todos os seguintes: sinais e sintomas de insuficiência cardíaca agudamente descompensada (ICAD), fração de ejeção do ventrículo esquerdo de 40% ou menos, sintomas de classe funcional II a IV da New York Heart Association e evidência clínica de congestão venosa necessitando de diuréticos intravenosos. Foram excluídos pacientes com cirrose hepática, aqueles em diálise na alta, aqueles que recusaram participação e aqueles com reinternação planejada para procedimentos eletivos, como revascularização ou ablação de arritmia.

O estudo foi realizado em 2 hospitais terciários da Região Sul do Brasil no período de setembro de 2021 a novembro de 2022 e foi aprovado pelos Comitês de Ética em Pesquisa de ambas as instituições. O consentimento informado por escrito foi obtido de todos os pacientes ou de seus representantes legais antes da inclusão.

### Desenho do estudo

Os pacientes receberam tratamento de cardiologistas e residentes em cardiologia que não estavam envolvidos no estudo, e os pesquisadores foram notificados no momento da alta hospitalar. Momentos antes da alta, após a obtenção do consentimento, um único operador (PR) realizou avaliações ultrassonográficas (escore VExUS e ultrassonografia pulmonar) e exames clínicos para avaliar o escore EVEREST. Os achados ultrassonográficos não foram divulgados ao médico assistente e não influenciaram o plano de tratamento dos pacientes.

### Coleta de dados e desfechos

Os dados de linha de base e de alta hospitalar foram extraídos de prontuários médicos eletrônicos. Coletamos dados demográficos (idade, sexo), histórico médico (comorbidades, histórico de internação por ICAD), classe NYHA na admissão, necessidade de ventilação não invasiva, vasodilatadores intravenosos e uso de inotrópicos durante a internação hospitalar. Foram registrados marcadores bioquímicos (hemoglobina, creatinina e sódio) e medicamentos utilizados na admissão e prescritos na alta, bem como a pressão arterial e a frequência cardíaca na alta. Também foram coletadas as últimas medidas disponíveis de avaliação ecocardiográfica, incluindo fração de ejeção do ventrículo esquerdo e presença de disfunção sistólica do ventrículo direito, definida como a presença de uma das seguintes: excursão sistólica do plano do anel tricúspide (TAPSE) < 16 mm, velocidade da onda sistólica do anel lateral tricúspide derivada do Doppler tecidual (S’) < 9,5 cm/s ou variação fracional da área (FAC) < 35%.

O desfecho primário foi um desfecho composto de readmissão ou visitas de emergência devido à ICAD (definida como admissão hospitalar para administração intravenosa de diuréticos para controlar o estado volêmico) dentro de 90 dias após a alta hospitalar. Os dados de seguimento foram coletados por meio de revisão dos prontuários hospitalares dos pacientes e entrevista telefônica. Para evitar viés, também foram excluídos os pacientes que faleceram durante o seguimento, pois o óbito era um evento concorrente à readmissão.

### Escore EVEREST

O escore EVEREST foi calculado como a soma das pontuações que variam de 0 a 3 atribuídas a 6 parâmetros clínicos: dispneia, ortopneia, fadiga, pressão venosa jugular, estertores e edema periférico, resultando em uma pontuação final que varia de 0 (sem congestão) a 18 (congestão clínica máxima).^10,11^ Na alta, um escore EVEREST de ≥ 2 foi interpretado como indicativo de congestão clínica residual.

### Avaliação ultrassonográfica

A avaliação ultrassonográfica foi realizada com M-TURBO, Fujifilm Sonosite Inc. Todos os pacientes foram colocados em decúbito dorsal com a cabeceira da cama a 30°. As imagens obtidas foram gravadas para avaliação subsequente.

### Escore VExUS

A avaliação do escore VExUS foi realizada utilizando o transdutor setorial e todos os achados do Doppler foram obtidos durante a fase expiratória final do ciclo respiratório. O diâmetro da porção intra-hepática da VCI foi inicialmente medido usando um corte longitudinal em posição subxifoide, a 2 cm da junção com a veia hepática.^15^ Caso não fosse obtida uma janela adequada, o transdutor era movido lateralmente para o lado direito do corpo sobre o fígado. O diâmetro máximo durante o ciclo respiratório foi registrado, e os pacientes com diâmetro da VCI menor que 2 cm foram classificados como tendo VExUS 0. Para pacientes com VCI pletórica (diâmetro ≥ 2 cm), o Doppler da veia hepática e da veia porta foi avaliado por meio de Doppler de onda pulsada. O Doppler da veia hepática foi interpretado como normal (S > D), levemente anormal (D > S) ou gravemente anormal (onda S reversa) com base nas ondas A, S e D, identificadas pelo Doppler de onda pulsátil. O Doppler da veia porta foi avaliado com base nas velocidades de pico (Vmax) e nadir (Vmin) durante o ciclo cardíaco, e a fração de pulsatilidade foi posteriormente calculada ([Vmax − Vmin] / Vmax) e interpretada da seguinte maneira: normal (fração de pulsatilidade < 0,3), levemente anormal (fração de pulsatilidade de 0,3 a < 0,5) ou gravemente anormal (fração de pulsatilidade ≥ 0,5). A interpretação do escore VExUS utilizada neste estudo não incluiu avaliação de veias intrarrenais, semelhante à abordagem utilizada por Bhardwaj et al.: 0 (VCI < 2 cm), 1 (VCI ≥ 2 cm e padrões Doppler normais), 2 (VCI ≥ 2 cm e pelo menos 1 alteração leve no Doppler) ou 3 (VCI ≥ 2 cm e pelo menos 1 anormalidade Doppler grave).^16^

### Ultrassonografia pulmonar

A avaliação ultrassonográfica pulmonar foi realizada usando um transdutor convexo com abordagem de exame de 8 zonas de 15 cm de profundidade. Cada pulmão foi dividido em 4 zonas, e uma zona era considerada positiva se fossem identificadas ≥ 3 linhas B.^17^ O número de zonas pulmonares positivas foi categorizado nos seguintes 3 grupos: 0 a 1 zona positiva, 2 a 3 zonas positivas e 4 a 8 zonas positivas.

### Interpretação da ultrassonografia pulmonar e do escore VExUS

PR e MMB avaliaram as imagens gravadas independentemente. Eles não tinham conhecimento dos dados clínicos e dos desfechos, e quaisquer divergências foram resolvidas por meio de discussão.

### Análise estatística

As variáveis contínuas são apresentadas como média ± desvio padrão ou mediana com intervalo interquartil de acordo com a normalidade dos dados, enquanto as variáveis categóricas são apresentadas como frequências e proporções. A normalidade dos dados foi avaliada pelo teste de Shapiro-Wilk. As diferenças entre os grupos na linha de base do estudo foram analisadas usando o teste t de Student não pareado ou o teste U de Wilcoxon-Mann-Whitney, dependendo dos pressupostos de normalidade. Foi utilizado o teste qui-quadrado para analisar as variáveis categóricas. Foram calculados sensibilidade, especificidade, valor preditivo positivo e valor preditivo negativo para o escore VExUS 2 ou 3 como uma análise post hoc. Todas as análises estatísticas foram realizadas utilizando o IBM SPSS Statistics, versão 20.0 (IBM Corp., Armonk, NY, EUA). A significância estatística foi estabelecida em p < 0,05.

## Resultados

### Características da amostra do estudo

Inicialmente incluímos 58 pacientes; no entanto, 9 indivíduos foram excluídos. Dois foram excluídos por reinternação planejada, especificamente, 1 para ablação de *flutter* atrial e 1 para cirurgia de revascularização do miocárdio. Um paciente necessitou de diálise na alta; 1 não forneceu consentimento e 5 participantes morreram durante o período de seguimento. A coorte final foi composta por 49 indivíduos, dentre os quais 11 (22,4%) apresentaram o desfecho primário.

As características basais da amostra do estudo estão descritas na Tabela 1. A população do estudo consistiu predominantemente de pacientes do sexo masculino com idade mediana de 60 anos. Quase metade dos pacientes apresentava cardiomiopatia isquêmica; 30% tinham histórico de internação por IC no último ano; 55% apresentavam disfunção ventricular direita associada, e dois terços encontravam-se em classe funcional IV da NYHA na admissão. Adicionalmente, 70% dos pacientes estavam recebendo pelo menos 2 dos 4 pilares da terapia médica para ICFEr orientada pelas diretrizes na admissão, o que aumentou para 90% na alta.

**Tabela 1:** – Características basais da população do estudo

Variáveis	Todos os pacientes (n=49)	Sem desfecho primário (n=38)	Com desfecho primário (n=11)	p
	n (%)*	n (%)*	n (%)*	
Idade, ano^†^	59,5 (47,3 - 69,0)	56,0 (46,5 - 70,0)	64,0 (57,0 - 69,0)	0,259
Sexo masculino	35 (71,4)	25 (65,8)	10 (90,9)	0,104
**Condições preexistentes**				
Hipertensão	32 (65,3)	23 (60,5)	9 (81,8)	0,191
Diabetes	15 (30,6)	10 (26,3)	5 (45,5)	0,225
Cardiomiopatia isquêmica	21 (42,9)	16 (42,1)	5 (45,5)	0,843
Fibrilação atrial	14 (28,6)	12 (31,6)	2 (18,2)	0,386
DRC	4 (8,2)	2 (5,3)	2 (18,2)	0,168
DPOC	7 (14,3)	6 (15,8)	1 (9,1)	0,576
Admissão por ICAD nos últimos 12 meses	16 (32,7)	10 (26,3)	6 (54,5)	0,079
**Classificação funcional da NYHA**				0,439
II	5 (10,2)	3 (7,9)	2 (18,2)	
III	15 (30,6)	13 (34,2)	2 (18,2)	
IV	29 (59,2)	22 (57,9)	7 (63,6)	
**Medicamentos na linha de base**				
Betabloqueador	36 (73,5)	26 (68,4)	10 (90,9)	0,137
IECA/BRA	36 (73,5)	27 (71,1)	9 (81,8)	0,476
Antagonista mineralocorticoide	22 (44,9)	15 (39,5)	7 (63,6)	0,156
INRA	3 (6,1)	3 (7,9)	-	0,336
Nitratos	8 (16,3)	6 (15,2)	2 (18,2)	0,850
Inibidor de SGLT2	4 (8,2)	4 (10,5)	-	0,261
Digitálicos	9 (18,4)	4 (10,5)	5 (45,5)	0,008
Diuréticos de alça	37 (75,5)	28 (73,7)	9 (81,8)	0,581
Tiazídicos	2 (4,1)	-	2 (18,2)	0,047
**Dados laboratoriais na linha de base**				
Hemoglobina (g/dL)^‡^	13,4 ± 2,0	13,6 ± 2,0	12,8 ± 2,1	0,291
Creatinina (mg/dL)^†^	1,2 (0,9-1,4)	1,1 (0,9-1,3)	1,3 (1,1-1,6)	0,122
Sódio (mmol/L)^†^	139,0 (136,0-141,0)	140,0 (136,8-142,0)	136,0 (132,0-139,0)	0,018
**Dados da internação**				
Fração de ejeção^‡^	24,2 ± 6,4	24,3 ± 6,2	24,0 ± 7,0	0,896
Disfunção ventricular direita	27 (55,1)	21 (55,3)	6 (54,5)	0,966
Ventilação não invasiva	6 (12,2)	6 (15,8)	-	0,159
Vasodilatador intravenoso	15 (30,6)	11 (28,9)	4 (36,4)	0,638
Uso de inotrópicos	5 (10,2)	3 (7,9)	2 (18,2)	0,321
Tempo de internação hospitalar (dias)^†^	15,0 (9,0-21,0)	14,5 (8,8-21,0)	15,0 (10,0-18,0)	0,548
**Medicamentos na alta**				
Betabloqueador	47 (95,9)	37 (97,4)	10 (90,9)	0,340
IECA/BRA	34 (69,4)	28 (73,7)	6 (54,5)	0,225
Antagonista mineralocorticoide	39 (79,6)	28 (73,7)	11 (100,0)	0,057
INRA	7 (14,3)	5 (13,2)	2 (18,2)	0,675
Nitratos	13 (26,5)	10 (26,3)	3 (27,3)	0,950
Inibidor de SGLT2	10 (20,4)	8 (21,1)	2 (18,2)	0,835
Digitálicos	19 (38,8)	15 (39,5)	4 (36,4)	0,852
Diuréticos de alça	46 (93,9)	35 (92,1)	11 (100,0)	0,336
Tiazídicos	10 (20,4)	5 (13,2)	5 (45,5)	0,019
**Sinais vitais na alta**				
Pressão arterial sistólica^†^	109,0 (94,5-119,0)	109,5 (94,8-118,5)	105,0 (93,0-120,0)	0,914
Frequência cardíaca^‡^	72,4 ± 12,9	74,1 ± 13,6	66,5 ± 8,0	0,027
**Achados laboratoriais na alta**				
Creatinina (mg/dL)^†^	1,1 (1,0-1,6)	1,1 (1,0 - 1,3)	1,6 (1,1-2,1)	0,028
Sódio (mmol/L)^†^	138,0 (134,0-139,0)	138,0 (135,8-139,0)	134,0 (133,0-138,0)	0,047

BRA: bloqueador do receptor da angiotensina II; DPOC: doença pulmonar obstrutiva crônica; DRC: doença renal crônica; ICAD: insuficiência cardíaca agudamente descompensada; IECA: inibidor da enzima conversora de angiotensina; INRA: inibidor da neprilisina e do receptor de angiotensina-neprilisina; NYHA: Associação do Coração de Nova York; SGLT2: cotransportador sódio-glicose-2. * Salvo indicação contrária. ^†^ Os valores são expressos como mediana (intervalo interquartil). ^‡^ Os valores são expressos como média ± desvio padrão.

Os pacientes que apresentaram o desfecho primário exibiram uma proporção significativamente maior de uso de digoxina e tiazídicos na admissão, juntamente com controle mais estrito da frequência cardíaca e menores concentrações plasmáticas de sódio. Na alta, também apresentaram concentrações plasmáticas de creatinina mais elevadas e continuaram a apresentar níveis plasmáticos de sódio mais baixos. Além disso, 45,5% dos pacientes (5 de 11) receberam prescrição de bloqueio sequencial de néfrons, envolvendo a administração de 3 classes de diuréticos, em contraste com apenas 10,5% (4 de 38) dos indivíduos sem o desfecho (p = 0,08).

### Escore EVEREST, ultrassonografia pulmonar, escore VExUS na alta e desfecho primário

Na alta, a maioria dos pacientes (53,1%) apresentava escore EVEREST ≥ 2; 38,7% tiveram pelo menos 2 quadrantes positivos na ultrassonografia pulmonar e aproximadamente um terço teve escore VExUS de 2 ou 3. A Tabela 2 mostra o escore EVEREST, ultrassonografia pulmonar, escore VExUS e desfecho primário.

**Tabela 2:** – Escore EVEREST, ultrassonografia pulmonar, escore VExUS e desfecho primário

Variável	Todos os pacientes (n=49)	Sem desfecho primário (n=38)	Com desfecho primário (n=11)	p
	n (%)*	n (%)*	n (%)*	
Escore EVEREST^†^	2,0 (1,0 - 3,0)	1,5 (1,0 - 3,0)	2,0 (1,0 - 3,0)	0,759
Escore EVEREST ≥ 2	26 (53,1)	19 (50,0)	7 (63,6)	0,425
**Número de quadrantes positivos na ultrassonografia pulmonar**				0,692
0-1	30 (61,2)	24 (63,2)	6 (54,5)	
2-3	13 (26,5)	9 (23,7)	4 (36,4)	
4-8	6 (12,2)	5 (13,2)	1 (9,1)	
**Escore VExUS**				
0	22 (44,9)	20 (52,6)	2 (18,2)	
1	10 (20,4)	7 (18,4)	3 (27,3)	0,131^‡^
2 ou 3	17 (34,7)	11 (28,9)	6 (54,5)	0,044^‡^

EVEREST: Efficacy of Vasopressin Antagonism in Heart Failure: Outcome Study with Tolvaptan; VExUS: ultrassonografia de excesso venoso. * Salvo indicação contrária. ^†^ Os valores são expressos como mediana (intervalo interquartil). ^‡^ Usando escore VExUS 0 como referência.

Embora não tenha havido diferença no escore EVEREST e no número de zonas pulmonares positivas entre pacientes com e sem o desfecho primário, os participantes com escore VExUS de 2 e 3 tiveram uma proporção significativamente maior do desfecho primário (6 de 17, 35,3%) ao comparar com pacientes com escore VExUS 0 com o desfecho (2 de 22, 9%, p = 0,044), como demonstrado na Figura Central.

Quando cada componente individual do VExUS foi avaliado isoladamente, apenas VCI > 2 cm apresentou diferença estatisticamente significativa, pois esteve presente em 81,8% daqueles com o desfecho primário (9 de 11) e em 47,4% daqueles sem o desfecho (18 de 38, p = 0,043). A avaliação isolada do Doppler da veia hepática e da pulsatilidade da veia porta não mostrou associação com o desfecho primário. Porém, entre todos os participantes com VCI > 2 cm (27 de 49, 55,1%), apenas 33,3% apresentaram o desfecho e, destes, 66,7% apresentaram pelo menos 1 alteração no Doppler (VExUS 2 ou 3). Em contrapartida, entre aqueles com VCI > 2 cm, mas sem parâmetros Doppler alterados (VExUS 1), 70% não experimentaram o desfecho.

### Escore VExUS 2 ou 3 na alta e desfecho primário

Na previsão de readmissão ou visitas de emergência relacionadas à IC, o escore VExUS de 2 ou 3 exibiu uma sensibilidade de 54,5% e uma especificidade de 71%, produzindo um valor preditivo positivo de 35,3% e um valor preditivo negativo de 84,4%.

## Discussão

Verificamos que um número significativo de pacientes hospitalizados por ICAD apresentou um escore VExUS de 2 ou 3 na alta hospitalar, o que foi associado a um maior risco de readmissão ou visitas de emergência relacionadas à IC em 90 dias.

Embora os médicos visem alcançar descongestão eficaz para melhorar a prontidão para a alta hospitalar na ICAD,^1^ existe um consenso limitado sobre o melhor método para avaliá-la. Além da avaliação clínica da congestão, a presença de linhas B na ultrassonografia pulmonar no momento da alta foi associada a maior risco de readmissão.^-2^ Em nosso estudo, entretanto, o escore EVEREST foi muito semelhante entre os grupos, e a congestão pulmonar avaliada pela ultrassonografia pulmonar não demonstrou correlação com o desfecho. Uma possível explicação para isso seria o momento do estudo, realizado durante a pandemia de SARS-CoV-2, uma condição associada à pneumonia, caracterizada por resultados ultrassonográficos que podem incluir linhas B. Essa situação poderia ter contribuído potencialmente para uma especificidade reduzida do método de detecção precisa de congestão e pode ter interferido na previsão do desfecho.

O escore VExUS, uma ferramenta de beira de leito desenvolvida recentemente, integra a análise Doppler do fluxo venoso. Sua curva de aprendizagem fácil, especialmente para quem tem experiência em ultrassom *point-of-care*, facilita sua integração na prática clínica. Em nosso estudo, os pacientes com escore VExUS de 2 ou 3 tiveram desfechos piores do que aqueles com escore VExUS de 0. É um desafio determinar se um escore VExUS alterado reflete congestão esplâncnica persistente, a gravidade da doença cardiovascular subjacente ou ambas, pois esses fatores podem estar interligados. No entanto, isso pode ser visto como uma vantagem do escore VExUS. A pulsatilidade da veia porta reflete mais a presença de congestão esplâncnica residual, o que é importante, uma vez que a redistribuição anormal do volume do reservatório esplâncnico pode contribuir para a descompensação.^2^ Por outro lado, o diâmetro da VCI e as veias hepáticas são mais sensíveis às condições cardíacas, e ambos os fatores podem desempenhar um papel no prognóstico de pacientes com ICAD. Nossos dados demonstraram que um escore VExUS de 0, ou, em outras palavras, uma VCI não pletórica, significa um grupo com probabilidade muito baixa de readmissão. Embora uma VCI > 2 cm tenha sido significativamente associada ao desfecho, quase metade dos pacientes com essa característica não apresentou o desfecho, destacando a baixa especificidade desse parâmetro isolado nessa população. Em contrapartida, uma VCI pletórica com padrões Doppler normais (VExUS 1) ou alterações isoladas no Doppler não apresentaram correlação significativa com o desfecho. Esses achados sugerem que a integração das medidas da VCI com os parâmetros Doppler no escore VExUS pode aumentar a precisão da avaliação da congestão sistêmica residual.

Estudos anteriores demonstraram o valor prognóstico da ultrassonografia do território esplâncnico na avaliação de pacientes com IC. O diâmetro da VCI na admissão, na alta hospitalar ou no seguimento mostrou associação com maior mortalidade e/ou maior risco de readmissão.^2^ A pulsatilidade da veia porta também tem sido associada a resultados desfavoráveis em estudos recentes. No estudo de Bouabdallaoui et al., embora a pulsatilidade da veia porta tenha sido associada a piores resultados, não melhorou a discriminação do escore EVEREST. Até o momento, apenas um estudo avaliou o papel do escore VExUS nesse cenário. Torres-Arrese et al. verificaram que o escore VExUS na alta hospitalar não foi útil para prever a readmissão, enquanto nosso estudo sugere que possa identificar pacientes com maior risco. A diferença entre os dois estudos pode ser atribuída às características dos pacientes. Especificamente, nosso estudo incluiu 100% de pacientes com ICFEr, em comparação com apenas 13,5% no estudo de Torres-Arrese et al. Além disso, apenas 16,3% dos pacientes tiveram escore VExUS 2 ou 3 na alta hospitalar no estudo de Torres-Arrese et al., indicando menor gravidade da doença ou terapia descongestiva mais eficaz, em contraste com 34,7% no nosso estudo. Outra explicação para essa diferença poderia ser a abordagem variada do VExUS empregada nos estudos. O estudo mencionado utilizou uma abordagem mais complexa e menos sensível, incorporando Doppler intrarrenal e exigindo 1 anormalidade Doppler grave para VExUS 2 e 2 ou mais padrões Doppler graves para classificação de VExUS 3.

Nosso estudo tem algumas limitações. Primeiramente, utilizamos um método de amostragem por conveniência, que contou com avisos de alta feitos pela equipe médica responsável para inclusão. Essa abordagem pode ter levado a perdas e contribuído para o tamanho relativamente pequeno da nossa amostra final, afetando potencialmente a generalização dos nossos resultados. Em segundo lugar, devido ao tamanho limitado da amostra, não foi possível realizar a análise de regressão logística, o que restringe a interpretação dos nossos resultados. Terceiro, nosso estudo focou em uma população específica com fração de ejeção ≤ 40%, e os resultados podem não ser aplicáveis a pacientes com fração de ejeção preservada. Além disso, a presença de um escore EVEREST elevado, indicando congestão clínica residual, juntamente com níveis mais baixos de sódio, valores mais elevados de creatinina e um uso mais frequente de terapia tripla diurética, indica a possibilidade de que os pacientes com o desfecho tiveram maiores desafios para alcançar a descongestão ou que apresentavam congestão refratária. Também sugere que seus médicos poderiam estar cientes desses fatores no momento da alta. Finalmente, embora o VExUS possa servir como uma ferramenta útil para detectar congestão residual e avaliar o risco de descompensação futura, permanece incerto se representa um fator modificável ou apenas um indicador da gravidade da doença e dos desfechos. Por outro lado, nosso estudo foi o primeiro a investigar o VExUS como ferramenta prognóstica na alta de pacientes com ICFEr e um dos esforços pioneiros para avaliar essa técnica em pacientes com IC, atendendo a uma necessidade na área. Embora esta abordagem tenha sido incorporada na prática clínica, ainda carece de evidências científicas robustas.

## Conclusões

Nosso estudo revelou que uma proporção significativa de pacientes admitidos por ICAD apresentou escore VExUS de 2 ou 3 no momento da alta hospitalar. Esses pacientes apresentavam maior risco de readmissões ou visitas de emergência devido à ICAD após 90 dias. Nosso estudo serve como gerador de hipóteses, introduzindo o escore VExUS como uma ferramenta potencial adicional na desafiadora avaliação multimodal da congestão residual e do risco de readmissão por IC ou visitas de emergências após a alta em pacientes com ICFEr. Pesquisas adicionais são necessárias para investigar o potencial do escore VExUS como alvo para orientar a terapia de descongestão e melhorar os desfechos de pacientes com ICAD.
