# Supplementary material for: Escore VExUS na Alta Hospitalar como Preditor de Readmissão em Pacientes com Insuficiência Cardíaca Agudamente Descompensada: Estudo de Coorte
Source: Arq Bras Cardiol. 2024 May 23;121(5):e20230745. [Article in Portuguese] doi: 10.36660/abc.20230745 (PMC11164437; doi:10.36660/abc.20230745)
Supplement: Translation

# Translation: VExUS Score at Discharge as a Predictor of Readmission in Patients with Acute Decompensated Heart Failure: A Cohort Study

**DOI:** 10.36660/abc.20230745i

**Authors:** Paulo Maciel Rinaldi, Marcos Frata Rihl, Márcio Manozzo Boniatti

**Affiliations:** 1 Brazil Universidade Federal do Rio Grande do Sul, Porto Alegre, RS – Brazil; 2 Brazil Faculdade de Medicina da Universidade do Vale do Rio dos Sinos (UNISINOS), São Leopoldo, RS – Brazil; 3 Brazil Hospital de Clínicas de Porto Alegre, Porto Alegre, RS – Brazil

**Keywords:** Heart Failure, Patient Readmission, Quality of Life

## Abstract

**Background:**

Residual venous congestion is a major contributor to readmission of patients with heart failure, and the venous excess ultrasound (VExUS) score is a potentially useful tool to evaluate systemic congestion.

**Objectives:**

To investigate the association between VExUS score before hospital discharge among patients with heart failure and the risk of readmission due to acute decompensated heart failure (ADHF) within 90 days after discharge.

**Methods:**

This prospective cohort study enrolled adults with signs and symptoms of ADHF, left ventricular ejection fraction of 40% or below (heart failure with reduced ejection fraction), New York Heart Association functional class II to IV symptoms, and clinical evidence of venous congestion necessitating intravenous diuretics. Just prior to discharge, we conducted VExUS score evaluation. The primary outcome was a composite endpoint of readmission or emergency visits due to ADHF within 90 days following hospital discharge. Statistical significance was set at p < 0.05.

**Results:**

The cohort comprised 49 individuals, 11 (22.4%) of whom experienced the primary outcome. At discharge, 34.7% of participants had VExUS score 2 or 3. Patients with VExUS 2 and 3 had a higher proportion of the primary outcome when compared with patients with VExUS of 0 (35.3% versus 9%, p = 0.044).

**Conclusions:**

A significant proportion of patients with heart failure with reduced ejection fraction admitted for ADHF presented clinical and ultrasound signs of residual congestion at discharge. Patients with VExUS score of 2 or 3 at the time of hospital discharge were found to be at higher risk of readmissions or emergency visits due to ADHF after 90 days.

## Introduction

Heart failure (HF) is a highly prevalent condition worldwide, affecting over 64 million people and accounting for a significant proportion of hospitalizations and readmissions.^1,2^ Unfortunately, up to 50% of patients are readmitted within 6 months after initial HF hospitalization,^3^ which can substantially affect their quality of life.^4^

Congestion-related signs and symptoms are among the most common causes of hospitalization for HF and subsequent readmissions,^5^ highlighting the significance of unresolved congestion following acute decompensated heart failure (ADHF) as a major contributor to higher readmission rates.^6^ Consequently, managing clinical congestion has long been one of the primary goals of hospitalization.^7^ However, registry data reveals that around 40% of patients are discharged despite persistent symptoms of HF.^8,9^ Moreover, elevated cardiac filling pressures may exist without clinical congestion, underscoring the role of subclinical hemodynamic abnormalities in HF pathophysiology. This reinforces the need for a comprehensive volume status evaluation to optimize volume management in ADHF patients.^10^

The Efficacy of Vasopressin Antagonism in Heart Failure: Outcome Study with Tolvaptan (EVEREST) score is a clinical tool used to assess congestion and guide decongestion therapy in patients with ADHF.^10,11^ However, current evidence suggests that traditional methods of assessing congestion, such as chest radiography and clinical assessment, may be limited in accuracy. Lung ultrasound (LUS) has emerged as a promising tool for assessing pulmonary congestion, with higher accuracy than traditional methods.^12,13^ In particular, the presence of B-lines on LUS has been shown to predict a higher risk of readmission or death in patients with HF.^14^ Another method of assessing systemic congestion is the venous excess ultrasonography (VExUS) score, which combines inferior vena cava (IVC) dilation and pulsed-wave Doppler of the hepatic, portal, and intrarenal veins.^15^ While the VExUS score has gained popularity in the evaluation of HF, its usefulness in guiding therapy or predicting outcomes remains uncertain. Therefore, this study aimed to investigate the association between VExUS score before hospital discharge among patients with heart failure with reduced ejection fraction (HFrEF) and the risk of readmission or emergency visits due to ADHF within 90 days after discharge.

## Methods

### Study population

This prospective cohort study with convenience sampling enrolled patients aged 18 years or older presenting with all of the following: signs and symptoms of acute decompensated heart failure (ADHF), left ventricular ejection fraction of 40% or below, New York Heart Association (NYHA) functional class II to IV symptoms, and clinical evidence of venous congestion necessitating intravenous diuretics. Patients with hepatic cirrhosis, those on dialysis at discharge, those who declined participation, and those with planned rehospitalization for elective procedures such as revascularization or arrhythmia ablation were excluded.

The study took place at 2 tertiary hospitals in the South Region of Brazil from September 2021 to November 2022 and was approved by the Research Ethics Committees of both institutions. Written informed consent was obtained from all the patients or their legal representatives prior to inclusion.

### Study design

Patients received treatment from cardiologists and cardiology fellows who were not involved in the study, and researchers were notified upon discharge. Just prior to discharge, after consent was obtained, a single operator (PR) conducted ultrasound evaluations (VExUS score and LUS) and clinical examinations to assess the EVEREST score. The ultrasound findings were not disclosed to the attending physician and did not influence the patient’s treatment plan.

### Data and outcome collection

Baseline and discharge data were extracted from electronic medical records. We collected demographic data (age, sex), medical history (comorbidities, history of ADHF admission), NYHA class at admission, need for noninvasive ventilation, intravenous vasodilators, and inotropes during hospitalization. Biochemical markers (hemoglobin, creatinine, and sodium) and medications used at admission and prescribed at discharge were recorded, as well as blood pressure and heart rate at discharge. Last available echocardiography evaluation measures were also collected, including left ventricular ejection fraction and presence of right ventricular systolic dysfunction, defined as one of the following: tricuspid annular plane systolic excursion < 16 mm, Doppler tissue imaging-derived tricuspid lateral annular systolic velocity wave (S’) < 9.5 cm/s, or fractional area change < 35%.

The primary outcome was a composite endpoint of readmission or emergency visits due to ADHF (defined as admission for inpatient intravenous administration of diuretics to manage volume status) within 90 days following hospital discharge. Follow-up data were collected through a review of the patient’s hospital records and a telephone interview. To avoid bias, patients who died during follow-up were also excluded, as death was a competing event for readmission.

### EVEREST score

The EVEREST score was calculated as the sum of scores ranging from 0 to 3 assigned to 6 clinical parameters: dyspnea, orthopnea, fatigue, jugular venous pressure, rales, and peripheral edema, resulting in a final score ranging from 0 (no congestion) to 18 (maximal clinical congestion).^10,11^ Upon discharge, an EVEREST score of ≥ 2 was interpreted as indicative of clinical residual congestion.

### Ultrasound assessment

Ultrasound assessment was performed using M-TURBO, Fujifilm Sonosite Inc. All patients were placed in a dorsal decubitus position with the head of the bed at 30°. Images obtained were recorded for subsequent evaluation.

### VExUS score

The VExUS score was assessed using a phased-array probe and all Doppler findings were obtained during the end-expiratory phase of the respiratory cycle. The diameter of the intrahepatic portion of the IVC was initially measured using a longitudinal view from a subxiphoid position, 2 cm from the junction with the hepatic vein.^15^ If an adequate view was not obtained, the probe was moved laterally to the right side of the body over the liver. The maximal diameter during the respiratory cycle was recorded, and patients with an IVC diameter less than 2 cm were classified as having VExUS 0. For patients with a plethoric IVC (diameter ≥ 2 cm), hepatic vein and portal vein Doppler were evaluated using pulsed-wave Doppler. Hepatic vein Doppler was interpreted as normal (S > D), mildly abnormal (D > S), or severely abnormal (reverse S) based on the A, S, and D waves identified using pulsed-wave Doppler. Portal vein Doppler was evaluated based on peak (Vmax) and nadir velocities (Vmin) during the cardiac cycle, and the pulsatility fraction was subsequently calculated ([Vmax − Vmin] / Vmax) and interpreted as follows: normal (pulsatility fraction < 0.3), mildly abnormal (pulsatility fraction 0.3 to < 0.5), or severely abnormal (pulsatility fraction ≥ 0.5). The VExUS score interpretation used in this study did not include an assessment of intrarenal veins, similar to the approach used by Bhardwaj et al.: 0 (IVC < 2 cm), 1 (IVC ≥ 2 cm and normal Doppler patterns), 2 (IVC ≥ 2 cm and at least 1 mild Doppler abnormality), or 3 (IVC ≥ 2 cm and at least 1 severe Doppler abnormality).^16^

### LUS

LUS assessment was performed using a curvilinear probe with an 8-zone examination approach of 15 cm depth. Each lung was divided into 4 zones, and a zone was considered positive if ≥ 3 B-lines were identified.^17^ The number of positive lung zones was categorized into the following 3 groups: 0 to 1 positive zone, 2 to 3 positive zones, and 4 to 8 positive zones.

### LUS and VExUS score interpretation

PR and MMB independently assessed the recorded images. They were blinded to the clinical data and outcomes, and any disagreements were adjudicated through discussion.

### Statistical analysis

Continuous variables are reported as mean ± standard deviation or median with interquartile range according to data normality, while categorical variables are presented as frequencies and proportions. Normality of the data was tested using the Shapiro-Wilk test. Differences between groups at baseline were analyzed using the unpaired Student’s t-test or Wilcoxon-Mann-Whitney U test, depending on normality assumptions. The chi-squared test was used to analyze categorical variables. The sensitivity, specificity, positive predictive value, and negative predictive value were calculated for VExUS score 2 or 3 as a post hoc analysis. All statistical analyses were conducted using IBM SPSS Statistics, version 20.0 (IBM Corp., Armonk, NY, USA). Statistical significance was set at p < 0.05.

## Results

### Study sample characteristics

We initially enrolled 58 patients; however 9 individuals were excluded. Two patients were excluded due to planned rehospitalization, specifically, 1 for atrial flutter ablation and 1 for coronary artery bypass graft surgery. One patient required dialysis at discharge; 1 declined to provide consent, and 5 participants died during follow-up period. The final cohort comprised 49 individuals, among whom 11 (22.4%) experienced the primary outcome.

The baseline characteristics of the study sample are described in Table 1. The study population predominantly consisted of male patients with a median age of 60 years. Nearly half of the patients had ischemic cardiomyopathy; 30% had a history of HF hospitalization during the last year; 55% had associated right ventricular dysfunction, and two thirds were in NYHA functional class IV upon admission. Seventy percent of patients were receiving at least 2 of the 4 pillars of guideline-directed medical therapy for HFrEF at admission, which increased to 90% at discharge.

**Table 1:** – Baseline characteristics of the study population

Variables	All patients (n=49)	Without primary outcome (n=38)	With primary outcome (n=11)	p
	n (%)*	n (%)*	n (%)*	
Age, years^†^	59.5 (47.3 - 69.0)	56.0 (46.5 - 70.0)	64.0 (57.0 - 69.0)	0.259
Male sex	35 (71.4)	25 (65.8)	10 (90.9)	0.104
**Preexisting conditions**				
Hypertension	32 (65.3)	23 (60.5)	9 (81.8)	0.191
Diabetes	15 (30.6)	10 (26.3)	5 (45.5)	0.225
Ischemic cardiomyopathy	21 (42.9)	16 (42.1)	5 (45.5)	0.843
Atrial fibrillation	14 (28.6)	12 (31.6)	2 (18.2)	0.386
CKD	4 (8.2)	2 (5.3)	2 (18.2)	0.168
COPD	7 (14.3)	6 (15.8)	1 (9.1)	0.576
Admission for ADHF in the last 12 months	16 (32.7)	10 (26.3)	6 (54.5)	0.079
**NYHA functional classification**				0.439
II	5 (10.2)	3 (7.9)	2 (18.2)	
III	15 (30.6)	13 (34.2)	2 (18.2)	
IV	29 (59.2)	22 (57.9)	7 (63.6)	
**Baseline medications**				
Beta blocker	36 (73.5)	26 (68.4)	10 (90.9)	0.137
CE inhibitor/ARB	36 (73.5)	27 (71.1)	9 (81.8)	0.476
Mineralocorticoid antagonist	22 (44.9)	15 (39.5)	7 (63.6)	0.156
ARNi	3 (6.1)	3 (7.9)	-	0.336
Nitrate	8 (16.3)	6 (15.2)	2 (18.2)	0.850
SGLT2 inhibitor	4 (8.2)	4 (10.5)	-	0.261
Digitalis	9 (18.4)	4 (10.5)	5 (45.5)	0.008
Loop diuretic	37 (75.5)	28 (73.7)	9 (81.8)	0.581
Thiazide	2 (4.1)	-	2 (18.2)	0.047
**Laboratory data at baseline**				
Hemoglobin (g/dL)^‡^	13.4 ± 2.0	13.6 ± 2.0	12.8 ± 2.1	0.291
Creatinine (mg/dL)^†^	1.2 (0.9-1.4)	1.1 (0.9-1.3)	1.3 (1.1-1.6)	0.122
Sodium (mmol/L)^†^	139.0 (136.0-141.0)	140.0 (136.8-142.0)	136.0 (132.0-139.0)	0.018
**Hospitalization data**				
Ejection fraction^‡^	24.2 ± 6.4	24.3 ± 6.2	24.0 ± 7.0	0.896
Right ventricular dysfunction	27 (55.1)	21 (55.3)	6 (54.5)	0.966
Noninvasive ventilation	6 (12.2)	6 (15.8)	-	0.159
Intravenous vasodilator	15 (30.6)	11 (28.9)	4 (36.4)	0.638
Inotrope use	5 (10.2)	3 (7.9)	2 (18.2)	0.321
Hospital LOS (days)^†^	15.0 (9.0-21.0)	14.5 (8.8-21.0)	15.0 (10.0-18.0)	0.548
**Discharge medications**				
Beta blocker	47 (95.9)	37 (97.4)	10 (90.9)	0.340
ACE inhibitor/ARB	34 (69.4)	28 (73.7)	6 (54.5)	0.225
Mineralocorticoid antagonist	39 (79.6)	28 (73.7)	11 (100.0)	0.057
ARNi	7 (14.3)	5 (13.2)	2 (18.2)	0.675
Nitrate	13 (26.5)	10 (26.3)	3 (27.3)	0.950
SGLT2 inhibitor	10 (20.4)	8 (21.1)	2 (18.2)	0.835
Digitalis	19 (38.8)	15 (39.5)	4 (36.4)	0.852
Loop diuretic	46 (93.9)	35 (92.1)	11 (100.0)	0.336
Thiazide	10 (20.4)	5 (13.2)	5 (45.5)	0.019
**Vital signs at discharge**				
Systolic blood pressure^†^	109.0 (94.5-119.0)	109.5 (94.8-118.5)	105.0 (93.0-120.0)	0.914
Heart rate^‡^	72.4 ± 12.9	74.1 ± 13.6	66.5 ± 8.0	0.027
**Laboratory findings at discharge**				
Creatinine (mg/dL)^†^	1.1 (1.0-1.6)	1.1 (1.0 - 1.3)	1.6 (1.1-2.1)	0.028
Sodium (mmol/L)^†^	138.0 (134.0-139.0)	138.0 (135.8-139.0)	134.0 (133.0-138.0)	0.047

ACE: angiotensin-converting enzyme; ADHF: acute decompensated heart failure; ARB: angiotensin II receptor blocker; ARNi: angiotensin receptor-neprilysin inhibitor; CKD: chronic kidney disease; COPD: chronic obstructive pulmonary disease; LOS: length of stay; NYHA: New York Heart Association; SGLT2: sodium-glucose co-transporter-2. * Unless otherwise stated. ^†^ Values are expressed as the median (interquartile range). ^‡^ Values are expressed as the mean ± standard deviation.

Patients who experienced the primary outcome exhibited a significantly higher proportion of digoxin and thiazide use at admission, along with more restricted heart rate control and lower plasma sodium concentrations. Upon discharge, they also showed higher plasma creatinine concentrations and continued to have lower plasma sodium levels. Furthermore, 45.5% of patients (5 out of 11) were prescribed sequential nephron blockade, involving the administration of 3 classes of diuretics, in contrast to only 10.5% (4 out of 38) of individuals without the outcome (p = 0.08).

### EVEREST score, LUS, VExUS score at discharge, and primary outcome

At discharge, the majority of patients (53.1%) had an EVEREST score ≥ 2; 38.7% had at least 2 positive quadrants on LUS, and approximately one third had a VExUS score of 2 or 3. Table 2 displays the EVEREST score, LUS, VExUS score, and primary outcome.

**Table 2:** – EVEREST score, LUS, VExUS score, and primary outcome

Variable	All patients (n=49)	Without primary outcome (n=38)	With primary outcome (n=11)	p
	n (%)*	n (%)*	n (%)*	
EVEREST score^†^	2.0 (1.0 - 3.0)	1.5 (1.0 - 3.0)	2.0 (1.0 - 3.0)	0.759
EVEREST score ≥ 2	26 (53.1)	19 (50.0)	7 (63.6)	0.425
**Number of positive quadrants on the LUS**				0.692
0-1	30 (61.2)	24 (63.2)	6 (54.5)	
2-3	13 (26.5)	9 (23.7)	4 (36.4)	
4-8	6 (12.2)	5 (13.2)	1 (9.1)	
**VExUS score**				
0	22 (44.9)	20 (52.6)	2 (18.2)	
1	10 (20.4)	7 (18.4)	3 (27.3)	0.131^‡^
2 or 3	17 (34.7)	11 (28.9)	6 (54.5)	0.044^‡^

EVEREST: Efficacy of Vasopressin Antagonism in Heart Failure: Outcome Study with Tolvaptan; LUS: Lung ultrasound; VExUS: venous excess ultrasonography. * Unless otherwise stated. ^†^ Values are expressed as the median (interquartile range). ^‡^ Using VExUS score 0 as reference.

While there was no difference in the EVEREST score and number of positive lung zones between patients with and without the primary outcome, participants with VExUS score of 2 and 3 had a significantly higher proportion of the primary outcome (6 out of 17, 35.3%) when comparing with patients with VExUS score of 0 with the outcome (2 out of 22, 9%, p = 0,044), as demonstrated in the Central Illustration.

When each individual component of VExUS was assessed in isolation, only IVC > 2 cm showed a statistically significant difference, as it was present in 81.8% of those with the primary outcome (9 out of 11) and in 47.4% of those without the outcome (18 out of 38, p = 0.043). The isolated assessment of the hepatic vein Doppler and the pulsatility of the portal vein showed no association with the primary outcome. However, among all participants with IVC > 2 cm (27 out of 49, 55.1%), only 33.3% experienced the outcome, and, of these, 66.7% had at least 1 Doppler abnormality (VExUS 2 or 3). In contrast, among those with IVC > 2 cm but without any altered Doppler parameters (VExUS 1), 70% did not experience the outcome.

### VExUS score 2 or 3 at discharge and primary outcome

In predicting HF-related readmission or emergency visits, the VExUS score of 2 or 3 exhibited a sensitivity of 54.5% and a specificity of 71%, yielding a positive predictive value of 35.3% and a negative predictive value of 84.4%.

## Discussion

We found that a significant number of patients hospitalized for ADHF exhibited a VExUS score of 2 or 3 at hospital discharge, which was associated with a higher risk of HF-related readmission or emergency department visits at 90 days.

While clinicians aim to achieve effective decongestion to improve discharge readiness in ADHF,^1^ there is limited consensus on the best method for evaluating it. In addition to the clinical assessment of congestion, the presence of B-lines on LUS at the time of discharge was associated with a greater risk of readmission.^14,1-2^ In our study, however, the EVEREST score was very similar between groups, and lung congestion evaluated with LUS did not demonstrate correlation with the outcome. One potential explanation for this was the timing of the study during the SARS-CoV-2 pandemic, a condition associated with pneumonia, marked by ultrasound findings that may include B-lines. This situation could have potentially contributed to a reduced specificity of the method for accurately detecting congestion and may have interfered with the prediction of the outcome.

The VExUS score, a recently developed bedside tool, integrates Doppler analysis of venous flow. Its shallow learning curve, especially for those with point-of-care ultrasound experience, facilitates its integration into clinical practice. In our study, patients with a VExUS score of 2 or 3 had poorer outcomes than those with a VExUS score of 0. It is challenging to determine whether an altered VExUS score reflects persistent splanchnic congestion, the severity of underlying cardiovascular disease, or both as these factors may be intertwined. However, this could be seen as an advantage of the VExUS score. Portal vein pulsatility is more reflective of the presence of residual splanchnic congestion, which is important since abnormal volume redistribution from the splanchnic reservoir may contribute to decompensation.^2^ On the other hand, the IVC diameter and hepatic veins are more sensitive to cardiac conditions, and both factors may play a role in the prognosis of patients with ADHF. Our data has demonstrated that a VExUS score of 0, or, in other words, a non-plethoric IVC, signifies a group with a very low likelihood of readmission. While an IVC > 2 cm was found to be significantly associated with the outcome, nearly half of the patients with this characteristic did not experience the outcome, highlighting the low specificity of this isolated parameter in this population. In contrast, a plethoric IVC with normal Doppler patterns (VExUS 1) or isolated Doppler alterations did not show a significant correlation with the outcome. These findings suggest that the integration of IVC measurements with the Doppler parameters in the VExUS score may enhance the accuracy of assessing residual systemic congestion.

Previous studies have demonstrated the prognostic value of splanchnic compartment ultrasound in evaluating patients with HF. IVC diameter at admission, at hospital discharge, or at follow-up have shown an association with higher mortality and/or a higher risk of readmission.^2-2^ Portal vein pulsatility has also been associated with poor outcomes in recent studies. In the study by Bouabdallaoui et al.,^9^ although portal vein pulsatility was associated with worse outcomes, it did not improve EVEREST score discrimination. To date, only one study has evaluated the role of VExUS score in this scenario. Torres-Arrese et al. found that the VExUS score at hospital discharge was not useful to predict readmission, while our study suggests that it can identify patients at higher risk. The difference between the two studies could be attributed to patient characteristics. Specifically, our study included 100% of patients with HFrEF, compared to only 13.5% in the study by Torres-Arrese. Moreover, only 16.3% of patients had a VExUS score 2 or 3 at hospital discharge in the study by Torres-Arrese, indicating either a lower severity of disease or more effective decongestive therapy, in contrast to 34.7% in our study. Another explanation for that difference could be the varied VExUS approach employed in studies. The aforementioned study utilized a more intricate and less sensitive approach, incorporating intrarenal Doppler and requiring 1 severe Doppler abnormality for VExUS 2 and 2 or more severe Doppler patterns for VExUS 3 classification.

Our study has several limitations. First, we utilized a convenience sampling method, which relied on discharge announcements made by the attending medical team for inclusion. This approach may have led to losses and contributed to the relatively small size of our final sample, potentially affecting the generalizability of our findings. Second, due to the limited sample size, we were unable to conduct logistic regression analysis, which restricts the interpretation of our results. Third, our study focused on a specific population with ejection fraction ≤ 40%, and the results may not be applicable to patients with preserved ejection fraction. Furthermore, the presence of an elevated EVEREST score, indicating residual clinical congestion, along with lower sodium levels, higher creatinine values, and a more frequent use of diuretic triple therapy hints at the possibility that patients with the outcome experienced greater challenges in achieving decongestion or had refractory congestion. It also suggests that their physicians may have been aware of these factors at the time of discharge. Finally, while VExUS can serve as a useful tool for detecting residual congestion and assessing the risk of future decompensation, it remains uncertain whether it represents a modifiable factor or merely an indicator of disease severity and outcomes. On the other hand, our study was the first to investigate VExUS as a prognostic tool in the discharge of patients with HFrEF and one of the pioneering efforts to assess this technique in patients with HF, addressing a need in the field. Although this approach has been incorporated into clinical practice, it still lacks robust scientific evidence.

## Conclusions

Our study revealed that a significant proportion of patients admitted for ADHF had a VExUS score of 2 or 3 at the time of hospital discharge. These patients were found to be at higher risk of readmissions or emergency visits due to ADHF after 90 days. Our study serves as a hypothesis generator, introducing the VExUS score as an additional potential tool in the challenging multimodal assessment of residual congestion and the risk of HF readmission or emergency department visits upon discharge in patients with HFrEF. Additional research is needed to investigate the potential of the VExUS score as a target for guiding decongestion therapy and improving the outcomes of patients with ADHF.
